# Regulation of FTO on PDCD5 mRNA stability to mediate neuron apoptosis in rats with hypoxic-ischemic brain damage

**DOI:** 10.1515/tnsci-2025-0394

**Published:** 2026-06-08

**Authors:** Taiyong Yin, Minshu Zou, Dali Liu, Cui Shao, Shuangshuang Li, Hui Yuan, Hongtao Xu

**Affiliations:** Department of Pediatrics, General Hospital of Central Theater Command of PLA, Wuhan, China

**Keywords:** hypoxic-ischemic brain damage, FTO, m6A, PDCD5, neuron, apoptosis

## Abstract

**Objectives:**

This study explored the mechanism of FTO in regulating neuron apoptosis in hypoxic-ischemic brain damage (HIBD) models via PDCD5.

**Methods:**

Neonatal SD rats were treated by unilateral common carotid artery ligation to establish HIBD model, while primary hippocampal neurons were induced by oxygen-glucose deprivation (OGD) to mimic the *in vitro* model. The expression levels of FTO and PDCD5 mRNA and proteins were assessed by qRT-PCR and Western blot, respectively. Cell apoptosis was evaluated by CCK-8 assay and flow cytometry. Furthermore, cerebral infarct area and neuronal damage in HIBD rats were determined by TTC and Nissl stainings, whereas apoptosis in brain tissue was examined by TUNEL staining. MeRIP assay was performed to detect the levels of m6A modification, and RIP assay to confirm their binding relationship.

**Results:**

HIBD rats had decreased FTO expression and elevated PDCD5 expression, accompanied by increased cerebral infarction and apoptosis. Overexpression of FTO inhibited neuronal apoptosis and alleviated HIBD progression. Additionally, knockdown of PDCD5 suppressed OGD-induced neuronal apoptosis in OGD-induced primary hippocampal neurons, while such effect was nullified by overexpression of FTO. FTO interacted with PDCD5 to influence the m6A modification level of PTBP1 and subsequently decrease its mRNA stability.

**Conclusions:**

Down-regulation of FTO expression in HIBD reduces the degradation of M6A-modified PDCD5 mRNA, thereby increasing PDCD5 protein expression. This elevation in PDCD5 promotes neuronal apoptosis, which exacerbates the progression of HIBD.

## Introduction

Hypoxic-ischemic brain damage (HIBD) is a common cause for death or mental retardation in newborns [1], pertaining to the perinatal asphyxia and serious damages the central nervous system [2]. With a incidence of 2.5 per 1,000 live births, HIBD is routinely treated with therapeutic hypothermia (TH) as the first effective therapy, but less than half of the treated children shows improvement, placing a heavy burden on families and society, as TH must be performed within the first 6 h of birth [3], 4]. Although it is well documented that the two of the major contributors to HIBD are diffuse perfusion deficit and global hypoxic insult to the brain [5], the precise underlying mechanism of HIBI remains far been fully understood, which hinders the proposal of an effective treatment approach and necessitates the need for further exploration on its pathophysiology.

N6-methyladenosine (m6A) modification of RNA plays a key role in RNA metabolism, whose dysregulation has been identified in multiple neurological diseases, including Parkinson’s disease (PD), ischemic stroke, and Alzheimer’s disease (AD) [6], 7]. In mammals, m6A is installed by the methyltransferase complex and can be removed by m6A demethylase, such as fat mass and obesity associated (FTO) [8]. FTO, which was highly expressed in neurons, can affect neurological function through the m6A modification of mRNA [9], 10]. For instance, FTO/IGF2BP2 was found to inhibit the activation of NLRP3 inflammasome in OGD/R-induced microglia via suppressing NLRP3 m6A level [11]. In hypoxic-ischemic neonatal rats, FTO can destabilize PTEN mRNA to regulate synaptic and cognitive impairment [6]. Studies supported that FTO can suppress neuronal ferroptosis in the context of cerebral I/R injury [12], but less information is available regarding whether and how FTO regulates neuron apoptosis in HIBD.

Programmed cell death protein 5 (PDCD5) is a strong candidate of apoptosis-regulated protein. [13]. PDCD5 was also found to be mutated or dysregulated in the brain of patients with AD [14]. In addition to that, a previous study uncovered the regulatory role of PDCD5 in apoptosis and autophagy after middle cerebral artery occlusion (MCAO) treatment in mice [15]. Overexpression of PDCD5 can enhance ischemic neuron damage in MCAO rats [16]. Current study identified the presence of several m6A binding sites in PDCD5. therefore, we hypothesize that FTO may regulate m6A-modified PDCD5 to mediate neuron apoptosis in hypoxic-ischemic (HI) conditions. To this end, this study aim to explore the possible effect and mechanism of FTO on HIBD using HIBD rat models and OGD-induced primary rat hippocampal neurons.

## Materials and methods

### Ethics statement

The pregnant Sprague Dawley (SD) rats, purchased from Hunan SJA Laboratory Animal Co., Ltd. (Changsha, China) were caged in light condition of 12 h per day and at temperature of 20–22 °C, without restriction to food and water. SD rats of postnatal day 7 (PND 7) were used as research subjects in this study. The experimental design was performed in accordance with the guidelines established by the National Institutes of Health (NIH) Guide for the Care and Use of Laboratory Animals. All animal experimental protocols were approved by the Animal Ethics Committee of Bestcell Model Biological Center (Approval number: BSMS-2025-03-10A).

### Establishment of HIBD models

On PND 7, rats were anesthetized with isoflurane (3 %) and subjected to unilateral common carotid artery ligation, followed by 2 h of exposure to 8 % oxygen and 92 % nitrogen at 37 °C to establish a HIBD model. A nebulizer was used to maintain the chamber humidity at 50–70 %. Rats with sham operation (sham group) were anesthetized to expose the left common carotid artery, but received either ligation or hypoxia. The rats were divided into sham, HIBD, HIBD+vector, and HIBD+FTO groups (n=6 per group). Rats in HIBD+vector and HIBD+FTO groups received *in vivo* lentiviral injection. On PND 3, rats were anesthetized and received intracerebroventricular transplantation of lentivirus, purchased from GeneChem (Shanghai, China). Lentivirus overexpressing FTO (or its negative control vector, 3 μL 7.5 × 10^5^ TU) was injected into the lateral ventricle at the speed of 1 μL/min. Using the Paxinos & Watson rat brain atlas, the stereotactic coordinates were defined relative to Bregma (0, 0, 0): anterior-posterior: −1.2 mm, mediolateral: −1.2 mm, dorsoventral: −2.2 mm. After maintaining at the injection site for 3 min, the microsyringe was carefully withdrawn. After suturing the rats’ scalps, povidone-iodine was applied for disinfection. Following the procedure, neonatal rats were placed back in their cages after fully regaining consciousness. Rats were euthanized 48 h after HI treatment. Brain tissues were then harvested for TTC staining, while others were fixed and embedded in paraffin for Nissl and TUNEL staining. Additionally, fresh brain samples were collected for gene and protein expression analyses.

### TTC staining

Coronal brain slice (100 µm-thickness) was incubated with 2,3,5-triphenyltetrazolium chloride (TTC, Sigma-Aldrich) at 37 °C for 15 min. Infarct area was measured using ImageJ software. The total infarct volume was determined by summing the infarct volumes of all five sections. The infarct volume was calculated using the following formula: [(normal cerebral hemisphere−non-infarct region in the affected cerebral hemisphere)]/total cerebral hemisphere area × 100 %.

### Nissl staining

Slices (5-µm) were stained with 1 % toluidine blue which was dissolved in distilled water, before staining with Nissl dye (Beyotime, Shanghai), fixation in distilled water, 20 min of preheating at 60 °C, and decoloring with 95 % ethanol. Neuronal damage in the CA1 region of the hippocampus was observed under a microscope across all groups.

### TUNEL staining

Based on the instructions of TUNEL staining assay kit (C1090, Beyotime Biotechnology), the sections were incubated with TUNEL reaction solution for 1 h, then washed with PBS, and stained with DAPI for 10 min to label nuclei. Sections were observed under a fluorescence microscope (Olympus, Tokyo, Japan), and six random fields in the CA1 region of the hippocampus were analyzed to calculate the percentage of TUNEL-positive cells.

### Cell culture and OGD treatment

Primary rat hippocampal neurons (RAT-iCELL-n600, iCell Bioscience Inc., Shanghai) were seeded at a density of 1 × 10^6^ cells per well onto poly-d-lysine-coated (Sigma-Aldrich, MO, USA) coverslips. Neurons were cultured in neurobasal medium (Sigma-Aldrich) supplemented with B27 (Thermo Fisher Scientific, MA, USA) and 2 mM l-glutamine (Thermo Fisher Scientific). Half of the medium was replaced every 3–4 days, and the neurons were maintained at 37 °C in a humidified atmosphere of 5 % CO_2_.

Based on previous studies [17], the culture medium was replaced with glucose-free DMEM. The primary hippocampal neurons were then incubated under conditions of 95 % N_2_, 5 % CO_2_, and 1 % O_2_ at 37 °C for 40 min. Post-hypoxia, the hippocampal neurons were transferred to neuronal culture medium and incubated at 37 °C with 5 % CO_2_ for 24 h.

After lentiviral infection for 72 h, the neurons were induced by OGD treatment. The lentiviral vectors were obtained from GeneChem (Shanghai GeneChem Co., Ltd., China), including a negative control for PDCD5 interference (sh-NC, 5 × 10^8^ TU/mL, sequence: 5′-CCG​GTC​TCC​GAA​CGT​GTC​ACG​TCT​CGA​GAC​GTG​ACA​CGT​TCG​GAG​ATT​TTT​G-3′), PDCD5 interference (sh-PDCD5, 3 × 10^8^ TU/mL, sequence: 5′-CCG​GTT​TTC​TTC​TGT​TGA​ATT​TCC​TCG​AGG​AAA​TTC​AAC​AGA​AGA​AAA​TTT​TTG-3′), a negative control for overexpression (vector, 2.5 × 10^8^ TU/mL, empty vector), FTO overexpression (FTO, 2.5 × 10^8^ TU/mL, full-length coding sequence of FTO) and PDCD5 overexpression (PDCD5, 6 × 10^8^ TU/mL, full-length coding sequence of PDCD5). The overexpression vector (GV358) and knockdown vector (GV248) were provided by GeneChem (Shanghai, China). Quality control of the lentiviruses was performed by the manufacturer, including assessments of viral titer and screening for mycoplasma, endotoxin, chlamydia, bacteria, and fungi.

### Quantitative real-time polymerase chain reaction (qRT-PCR)

Total RNAs were extracted using Trizol (Invitrogen, CA, USA) and subjected to reverse transcription into cDNA using a reverse transcription kit (Beyotime). Amplification was performed on the ABI 7900 system (ABI, USA) using Ultra SYBR mixture (CW2601, CWBIO). The relative expression of target genes was calculated using 2^−ΔΔCt^ method with β-actin as the internal control. The primer sequences are listed below: PDCD5-Forward: AGT​ATC​TTA​GCC​CAA​GTT​CTG​GA, PDCD5-Reverse: TAT​CAA​ACC​TTG​TTC​TGA​CAC​CTT​C; FTO-Forward: ACT​TCA​TGG​ATC​CTC​AGA​AGA​TG, FTO-Reverse: TTC​GCA​GCT​ATA​GCT​GTA​CAC​TG; β-actin-Forward: CTG​AGA​GGG​AAA​TCG​TGC​GT, β-actin-Reverse: CCA​CAG​GAT​TCC​ATA​CCC​AAG​A.

### Western blot

Proteins extracted from cells and hippocampal tissues using RIPA lysis buffer (P0013B, Beyotime) were quantified using a BCA protein assay kit (23225, Thermo Fisher Scientific). The protein separation was performed using 10 % sodium dodecyl sulfate-polyacrylamide gel electrophoresis (SDS-PAGE) and the protein was subsequently transferred onto PVDF membranes (Millipore Corporation, MA, USA) before overnight incubation at 4 °C with primary antibodies against PDCD5 (PA5-20682, Invitrogen; 1:1000) or FTO (ab280081, Abcam; 1:1000) or β-actin (ab8227, Abcam; 1:2000), and further interaction with goat anti-rabbit secondary antibody (ab205718, Abcam; 1:10000). After incubation with enhanced chemiluminescence (ECL) reagent, membranes were imaged using a GE Amersham Imager 600 (AI600, IL, GE Healthcare). The grayscale intensity of each protein band was quantified using ImageJ software (version 1.8.0).

### Cell counting kit-8 (CCK-8) assay

Cells were seeded into 96-well plates (2 × 10^3^ cells per well, 100 µL per well) and incubated at 37 °C in a 5 % CO_2_ incubator. Subsequently, 10 µL of CCK-8 reagent (NU679, DOJINDO, Japan) was added per well, followed by incubation at 37 °C with 5 % CO_2_ for another 4 h. A microplate reader from Heales (MB-530, Shenzhen, China) was used to detect the absorbance at 450 nm.

### Flow cytometry

Cells were detached using ethylenediaminetetraacetic acid (EDTA) free trypsin and centrifuged at 1,000 rpm for 5 min. The collected pellets were resuspended, washed with PBS, and centrifuged at 1,000 rpm for 5 min. Cell apoptosis was detected using an Annexin V-FITC/PI Double Staining Kit (40302ES20, Yeasen Biotech Co., Ltd., China) according to the manufacturer’s instructions. After being resuspended in 500 µL binding buffer, cells were incubated with 5 µL Annexin V-FITC. Subsequently, 5 µL propidium iodide (PI) was added, gently mixed, and incubated for 10 min in the dark. Data acquisition was performed using a flow cytometer. First, a loose gate was set to exclude cell debris in the lower-left corner. Next, a singlet gate was applied to remove doublets deviating from the diagonal. Fluorescence compensation was then adjusted based on the untreated negative control, followed by the setting of quadrant gates for analysis.

### m6A immunoprecipitation (MeRIP)-qPCR

MeRIP procedure was performed according to instructions from a Magna MeRIP™ m6A kit (#17-10,499, Merck Millipore, MA). The purified mRNA was first digested with DNase I, fragmented into 100 nt, and incubated at 94 °C. Fragmentation was halted with a stop buffer and the RNA was collected through ethanol precipitation. Meanwhile, 12 μg of anti-m6A antibody was pre-incubated with 50 μL of beads in IP buffer (150 mM NaCl, 0.1 % NP-40, 10 mM Tris-HCl, pH 7.4) at room temperature for 1 h. Afterward, 6 μg of fragmented mRNA was introduced into the antibody-bead mixture and incubated at 4 °C for 4 h on a rotator. Following thorough washing, the immunoprecipitated mixture was digested with high concentration of proteinase K. The bound RNAs were extracted using the phenol-chloroform method and ethanol precipitation before qPCR analysis. qPCR was performed to assess m6A modification in PDCD5 using specific primers (Forward: TGG​ACA​GCT​AAG​TGG​GAA​GGT, Reverse: GTA​AAC​ATT​TAC​AAG​GTG​GTG​ACT​G) Additionally, all potential m6A sites in PDCD5 were identified using SRAMP (http://www.cuilab.cn/sramp).

### RNA stability assay

Primary hippocampal neurons were collected and seeded into 12-well plates. RNA transcription was inhibited using Actinomycin D (5 μg/mL, Cell Signaling Technology, USA). Cells were collected at designated time points (0, 3, and 6 h) to evaluate mRNA degradation. Relative levels of PDCD5 mRNA were analyzed by qRT-PCR to assess the RNA stability.

### Statistical analysis

Statistical analysis was performed using GraphPad Prism 8.0 software (GraphPad Software, USA). All data were expressed as mean ± standard deviation (SD). All quantitative and statistical analyses were performed by researchers blinded to the experimental groupings. The sample size for all animal experiments was n=6 per group, and all cell-level experiments were repeated independently at least three times. For data following a normal distribution, comparisons between two groups were performed using Student’s *t*-test, while comparisons among multiple groups were conducted using one-way or two-way analysis of variance (ANOVA), followed by Tukey’s multiple comparisons test for post hoc analysis. Differences with a p-value <0.05 were considered statistically significant.

## Results

### Knockdown of PDCD5 suppressed OGD-induced neuronal apoptosis

**Figure 1:**
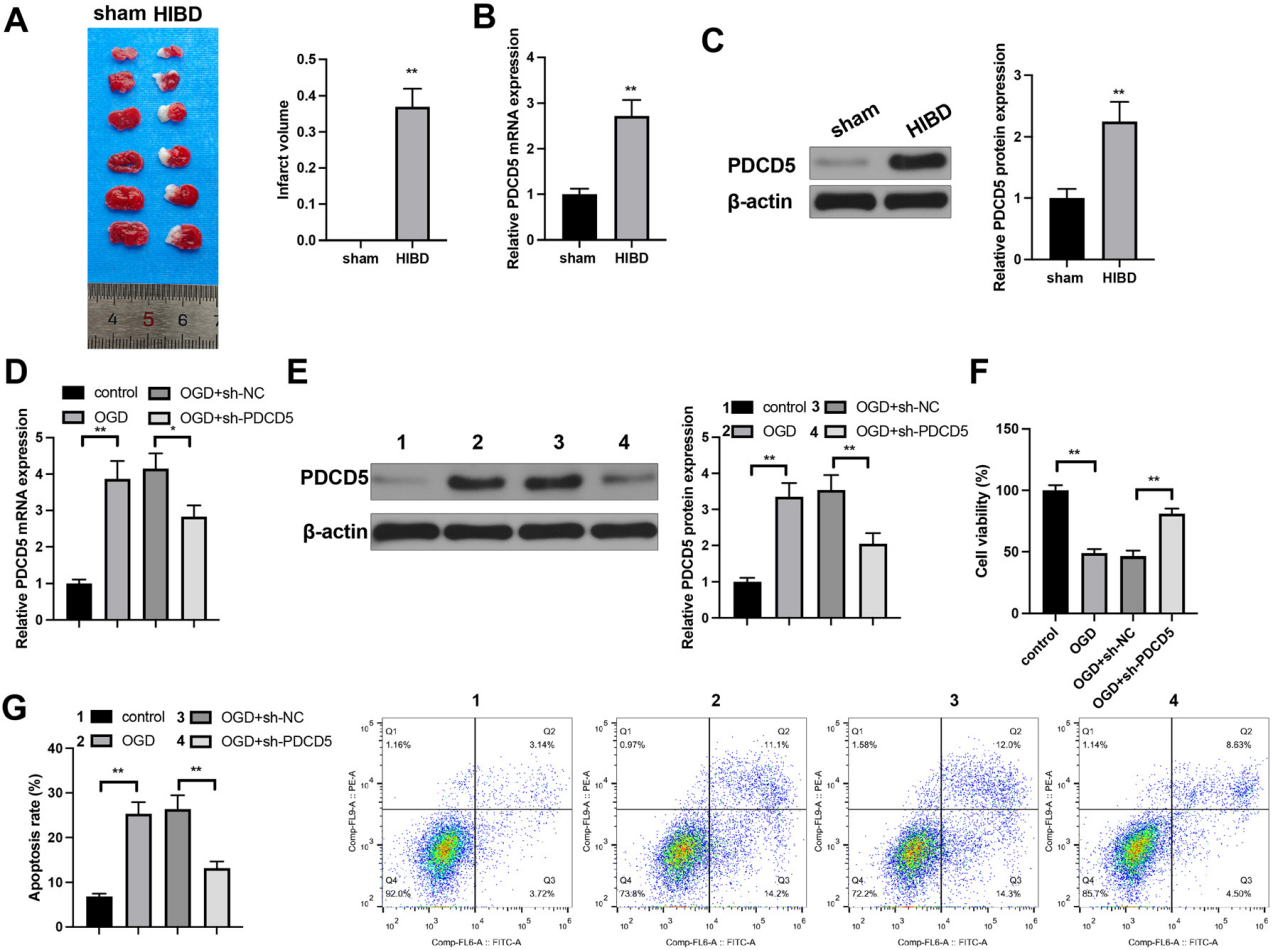
PDCD5 knockdown suppress OGD induced neuron apoptosis. (A) TTC staining detected infarct area in brain tissues of rats in HIBD and sham groups, n=6; (B) qRT-PCR detected PDCD5 mRNA expression in hippocampal tissues of rats in HIBD and sham groups, n=6; (C) western blot analysis evaluated PDCD5 protein expression in hippocampal tissues of rats in HIBD and sham groups, n=6; (D) qRT-PCR detected PDCD5 mRNA expression in primary hippocampal neurons after OGD treatment and PDCD5 knockdown, n=3; (E) western blot analysis evaluated PDCD5 protein expression in primary hippocampal neurons after OGD modeling and PDCD5 knockdown, n=3; (F) CCK-8 assay assessed hippocampal neuron viability after OGD modeling and PDCD5 knockdown, n=3; (G) flow cytometry quantified apoptosis rates in hippocampal neurons after OGD modeling and PDCD5 knockdown, n=3. Panel A–C was analyzed using Student’s *t*-test and analysis for panel D–G was determined by one-way ANOVA followed by Tukey’s multiple comparisons test. *p<0.05; **p<0.01.

SD rats at PND 7 were induced for HIBD model establishment. TTC staining verified the infarct area in HIBD group was much larger than that in sham group (Figure 1A; groups: sham and HIBD). Detection on PDCD5 expression levels shown that compared with sham group, HIBD group had elevated PDCD5 expression (Figure 1B and C; groups: sham and HIBD).

Neurons were transfected with sh-PDCD5 or sh-NC and then induced for OGD treatment to establish HIBD *in vitro* cellular models. qRT-PCR and western blot demonstrated elevated PDCD5 mRNA and protein levels in OGD group in contrast to control group, and reduced PDCD5 mRNA and protein levels in OGD+sh-PDCD5 group in contrast to OGD+sh-NC group. CCK-8 assay was performed to evaluate cell viability. The results demonstrated that, compared with the sham group, cell viability in the OGD group was significantly decreased; conversely, compared with the OGD+sh-NC group, cell viability in the OGD+sh-PDCD5 group was significantly increased (Figure 1F). Flow cytometry results found higher apoptosis rate in OGD group and lower apoptosis rate in OGD+sh-PDCD5 group, when respectively compared with control and OGD+sh-NC groups (Figure 1G).

### FTO mediates PDCD5 expression via m6A modification

**Figure 2:**
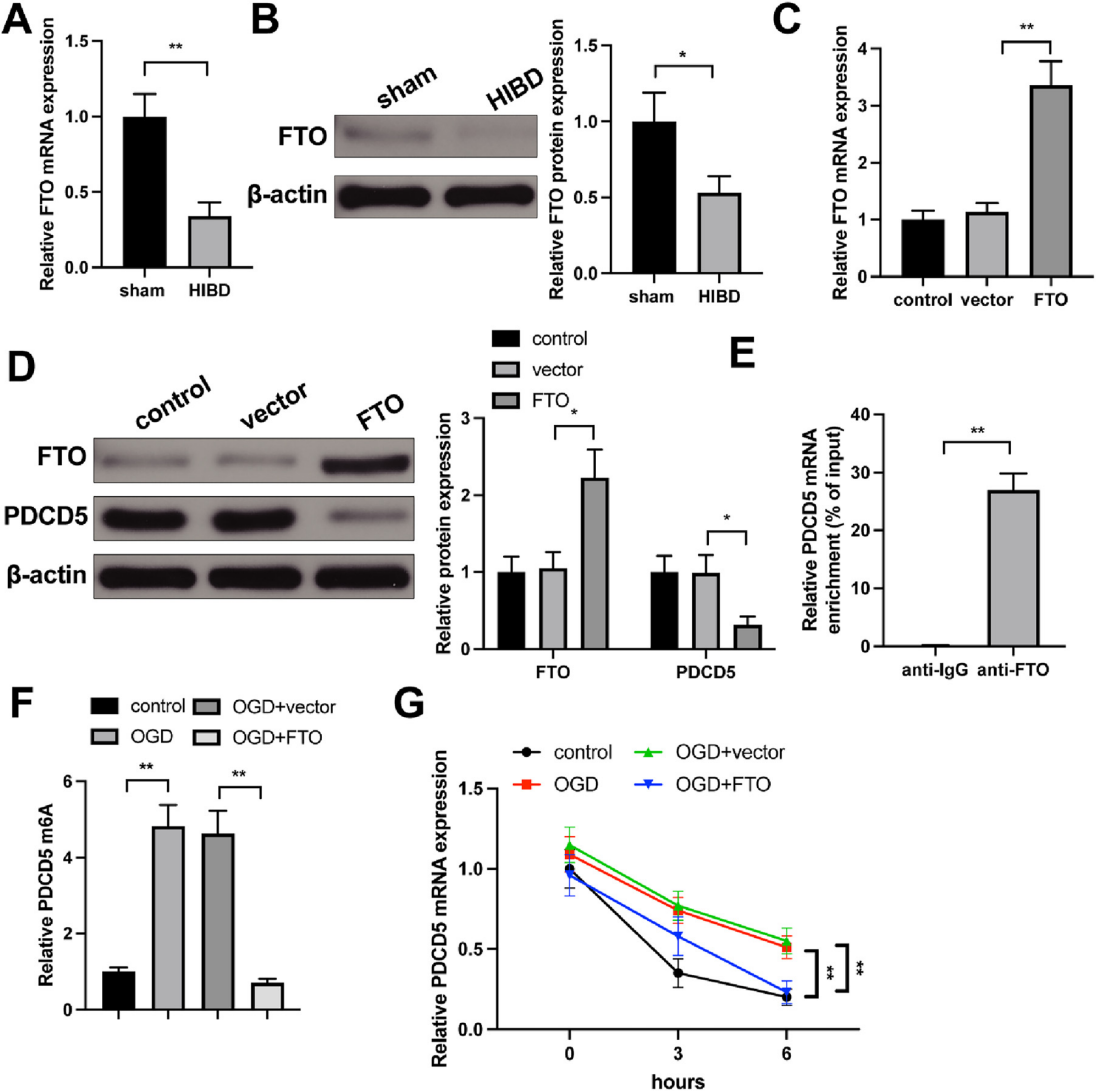
FTO mediates PDCD5 expression via m6A modification. (A) FTO mRNA level in hippocampal tissues of rats in HIBD and sham groups was detected by qRT-PCR, n=6; (B) FTO protein level in hippocampal tissues of rats in HIBD and sham groups was detected by western blot, n=6; (C–D) primary hippocampal neurons were transfected with FTO-overexpressing lentivirus. qRT-PCR and western blot both verified the successful overexpression of FTO and the effect of FTO overexpression on protein expression level of PDCD5 in hippocampal neurons, n=3; (E) RIP assay confirmed the binding of PDCD5 and FTO in hippocampal neurons, n=3; (F) after OGD treatment and FTO overexpression, PDCD5 m6A levels in hippocampal neurons was detected by MeRIP assay, n=3; (G) after OGD treatment and FTO overexpression, PDCD5 mRNA degradation rate was measured by Actinomycin D assay, n=3. Panel A, B and E was analyzed using student’s *t*-test and analysis for panel C, D, and F was determined one-way ANOVA followed by Tukey’s multiple comparisons test; panel G was analyzed by two-way ANOVA followed by Tukey’s multiple comparisons test. *p<0.05; **p<0.01.

Detection on FTO expression *in vivo* models found downregulated mRNA and protein expression of FTO in HIBD group (Figure 2A and B).

Bioinformatics analysis indicated that FTO could bind to PDCD5 (Supplementary Figure 1A), and multiple m6A binding sites were also identified on PDCD5 (Supplementary Figure 1B). Overexpression of FTO in hippocampal neurons led to successful FTO overexpression and reduced PDCD5 expression (Figure 2C and D). RIP assay confirmed the interaction between PDCD5 mRNA and FTO protein in hippocampal neurons (Figure 2E). Detection on m6A level by MeRIP assay found an increased m6A modification level on PDCD5 mRNA in the OGD group compared to the control group, while overexpression of FTO significantly reduced m6A modification levels on PDCD5 mRNA (Figure 2F). Furthermore, qRT-PCR analysis on hippocampal neurons treated with Actinomycin D revealed that FTO overexpression significantly decreased PDCD5 mRNA stability (Figure 2G).

### FTO downregulates PDCD5 expression to inhibit OGD-induced neuron apoptosis

**Figure 3:**
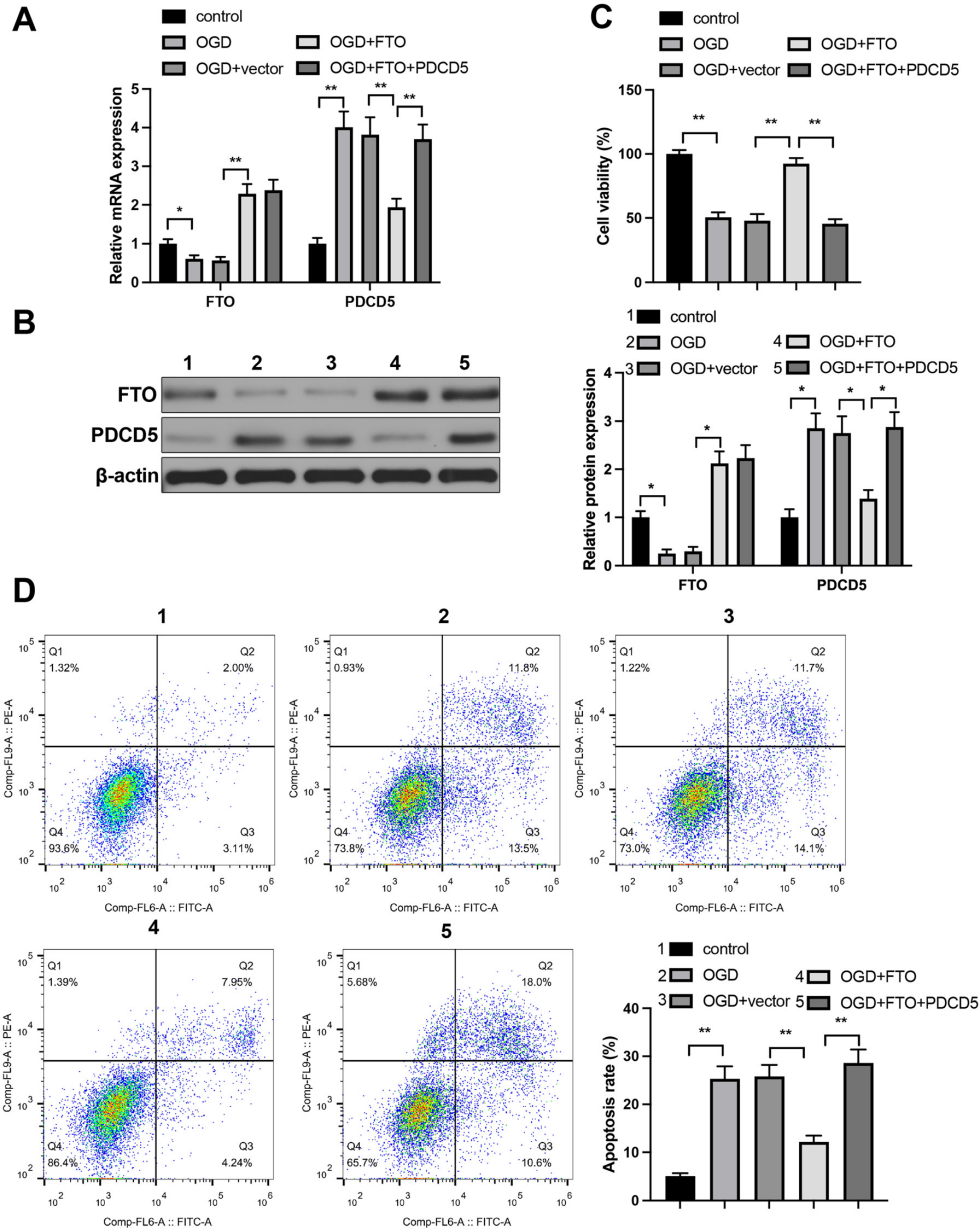
FTO inhibits OGD-induced neuron apoptosis via mediating PDCD5 expression. Primary hippocampal neurons were transfected with FTO and/or PDCD5 lentivirus, followed by the establishment of an OGD model, to investigate the effects of the FTO/PDCD5 axis on OGD-induced neuronal injury. (A) FTO and PDCD5 mRNA levels in hippocampal neurons were detected by qRT-PCR, n=3; (B) FTO and PDCD5 protein levels in hippocampal neurons were detected by western blot, n=3; (C) cell viability was assessed by CCK-8 assay, n=3; (D) apoptosis rate detected by flow cytometry, n=3. Panel A–D was analyzed using one-way ANOVA followed by Tukey’s multiple comparisons test. *p<0.05; **p<0.01.

The expression levels of FTO and PDCD5 were measured using qRT-PCR and western blot The results showed that, compared with the control group, FTO expression was downregulated while PDCD5 expression was upregulated in the OGD group. Relative to the OGD+vector group, the OGD+FTO group exhibited an increase in FTO levels and a concomitant decrease in PDCD5 levels. Furthermore, PDCD5 expression was significantly upregulated in the OGD+FTO+PDCD5 group compared with the OGD+FTO group (Figure 3A and B). Measurement on cell viability and apoptosis shown that cell viability was suppressed and apoptosis rate were increased in OGD and OGD+FTO+PDCD5 groups, when respectively compared with that in control and OGD+FTO group (Figure 3C and D). However, elevated cell viability and suppressed apoptosis rate was found in OGD+FTO group, in contrast to OGD+vector group (Figure 3C and D).

### Overexpression of FTO can suppress neuron apoptosis to attenuate HIBD

**Figure 4:**
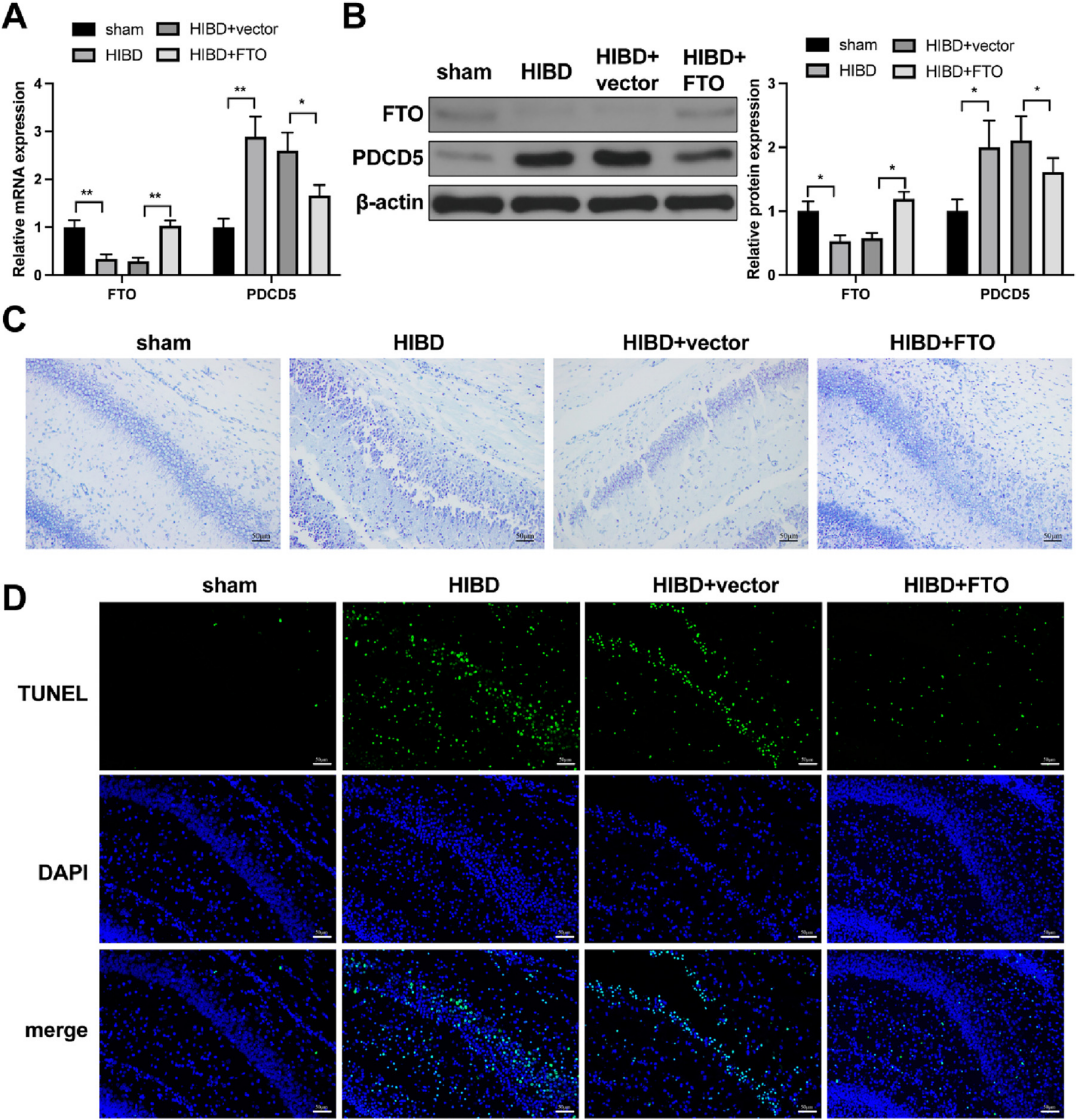
Overexpression of FTO can suppress neuron apoptosis to attenuate HIBD. Neonatal rats were transduced with FTO-overexpressing lentivirus, followed by the establishment of a HIBD model, to investigate the functional role of FTO *in vivo*. (A) FTO and PDCD5 mRNA levels in the brain tissues of rats in each group were detected by qRT-PCR, n=6; (B) FTO and PDCD5 protein levels in the brain tissues of rats in each group were detected by western blot, n=6; (C) Nissl staining evaluated neuronal damage in rats in each group, n=6; (D) neuron apoptosis in the brain tissues of rats in each group was assessed by TUNEL staining, n=6. Panel A–B was analyzed using one-way ANOVA followed by Tukey’s multiple comparisons test. *p<0.05; **p<0.01.

qRT-PCR and Western blot analysis showed that, compared with the sham group, FTO expression was decreased and PDCD5 expression was increased in hippocampal tissues of the HIBD group. Conversely, compared with the HIBD+vector group, FTO expression was elevated and PDCD5 expression was reduced in the HIBD+FTO group (Figure 4A and B). Nissl staining was performed to evaluate neuronal damage. In the HIBD group, a significant reduction in Nissl bodies was observed compared with the sham group, indicating increased neuronal injury. Conversely, the HIBD+FTO group showed an increase in Nissl bodies compared to the HIBD+vector group, suggesting that FTO treatment alleviated neuronal damage (Figure 4C). TUNEL staining revealed increased numbers of TUNEL-positive cells in hippocampal tissues of the HIBD group compared with the sham group. Furthermore, the number of TUNEL-positive cells was reduced in the HIBD+FTO group compared with the HIBD+vector group (Figure 4D).

## Discussion

This study aimed to investigate the potential regulatory role of FTO in HIBD using neonatal SD rats and OGD-induced primary neurons as preclinical models. The key findings of this study demonstrate that: (1) FTO is downregulated, whereas PDCD5 is increasingly expressed in these HIBD models; (2) knockdown of PDCD5 suppresses OGD-induced neuron apoptosis; (3) FTO mediates PDCD5 expression via m6A modification; and (4) FTO overexpression enhances PDCD5 mRNA degradation, thereby mitigating OGD-induced apoptosis. Conclusively, decreased FTO expression may contribute to HIBD progression by stabilizing m6A-modified PDCD5 and promoting neuron apoptosis. However, as these findings are derived strictly from preclinical animal and cell-based systems, their direct relevance to human neonatal HIBD remains to be fully validated in clinical settings.

PDCD5, formerly known as TF-1 cell apoptosis-related gene 19 (TFAR19), is an apoptosis-accelerating protein which can promote cell apoptosis in various cell types [18]. Increasing evidence supported PDCD5 was downregulated in different tumor cells, including hepatocellular carcinoma, and esophageal squamous cell carcinoma, whose overexpression contributed to increased apoptosis or pyroptosis in tumor cells [19], [20], [21]. In this study, up-regulated PDCD5 expression was detected in HIBD rat models and suppression on PDCD5 expression led to increased cell viability and decreased apoptosis, which was consistent with previous studies, highlight the regulatory role of PDCD5 on neuron apoptosis.

Bioinformatics analysis found the binding sites of FTO with PDCD5, as well as the presentation of m6A modification sites on PDCD5. Therefore, it is reasonably to speculate that FTO may regulate PDCD5 expression in HIBD models via m6A modification. The m6A modification regulates the stability and translation efficiency of mRNA, and its dynamic regulation involves an interplay among m6A methyltransferases (writers), demethylases (erasers), and m6A-binding proteins (readers) [22]. FTO is a well-documented m6A eraser and responsible for demethylating the internal m6A and cap m6Am in mRNA [23], 24]. Knockdown of FTO increases m6A modification levels, whereas its overexpression reduces m6A levels [25]. MeRIP in this study shown overexpression of FTO can decrease the mRNA expression of PDCD5, which was further supported by Actinomycin D assay reporting reduced PDCD5 mRNA stability in neurons transfected with FTO overexpression lentivirus. Previous study reported that FTO can suppress the m6A methylation of Gpr177 and lead to GRP177 overexpression, thereby regulating oxidative stress in neuropathic pain rat models [26]. Similarly, overexpression of FTO in HIBD models was found to decrease PDCD5 mRNA stability and decrease PDCD5 mRNA expression. Detection on FTO expression pattern found FTO expression was decreased whereas PDCD5 expression was elevated in OGD-induced neurons. The implication of FTO on neuronal development and pathogenesis of multiple central nervous system disorders has been reported in several studies. For instance, silencing FTO in neurons has been found to alleviate cognitive impairments in AD mouse models [27]. Observations in spinal cord injury mouse models also found that FTO upregulation ameliorated OGD-induced neuronal apoptosis [28]. In this study, rescue experiments on primary hippocampal neurons revealed that FTO overexpression regulates PDCD5 expression to increase cell viability and suppress neuron apoptosis, relieving the HIBD progression. Validation on HIBD rat models shown FTO was decreasingly expressed in hippocampal tissues of HIBD rats and overexpression of FTO can suppress the number of TUNEL-positive cells, underscoring the protective role of FTO overexpression against neuron apoptosis in HIBD rat models. Collectively, *in vivo* and *in vitro* experiments provide preliminary evidence for the potential regulation of the FTO/PDCD5 axis in HIBD models.

In conclusion, our findings demonstrate that FTO is down-regulated in HIBD rat models, which leads to increased m6A modification and expression of PDCD5, subsequently promoting neuron apoptosis and aggravating HIBD progression. These results identify the FTO/PDCD5 axis as a potential regulatory mechanism in HIBD pathogenesis. However, it should be noted that PDCD5 typically functions as an apoptosis enhancer, rather than a direct inducer [18], and the downstream targets of the FTO/PDCD5 axis remain to be fully elucidated. Furthermore, as these data are derived from preclinical animal and cell models, they represent preliminary evidence. Additional validation in higher species and clinical settings is warranted before these findings can be extrapolated to human neonatal HIBD.

Looking forward, future research should focus on three key areas. First, utilizing high-throughput sequencing such as RIP-seq or MeRIP-seq will be crucial to map the full spectrum of m6A-modified targets downstream of FTO in HIBD. Second, clinical studies measuring FTO and PDCD5 levels in the blood or cerebrospinal fluid of neonates with HIBD could validate the diagnostic and prognostic value of this axis. Finally, developing small-molecule activators of FTO or targeted inhibitors of the FTO/PDCD5 interaction may offer a novel therapeutic strategy for mitigating HIBD. These directions will be vital to transition from bench findings to potential bedside applications and to provide a more comprehensive therapeutic perspective on HIBD pathogenesis.

## Supplementary Material

Supplementary Material

Supplementary Material
