# Supplementary figures and images for: Regulation of FTO on PDCD5 mRNA stability to mediate neuron apoptosis in rats with hypoxic-ischemic brain damage
Source: Transl Neurosci. 2026 Jun 8;17(1):20250394. doi: 10.1515/tnsci-2025-0394 (PMC13241177; doi:10.1515/tnsci-2025-0394)

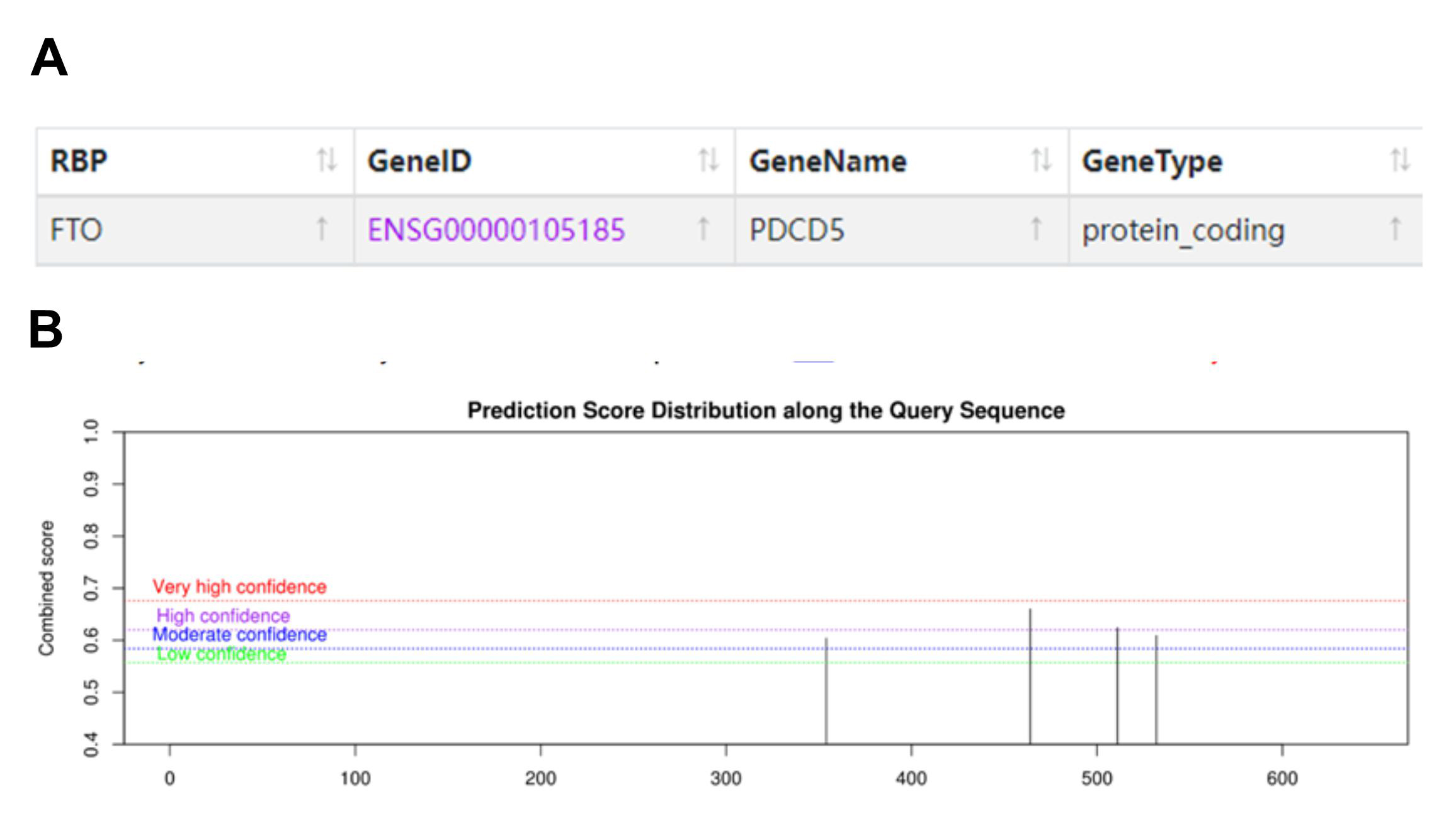

Supplement: Supplementary file 1 — Supplementary Material [file j_tnsci-2025-0394_suppl_001.jpg]
