# Supplementary material for: Regulation of FTO on PDCD5 mRNA stability to mediate neuron apoptosis in rats with hypoxic-ischemic brain damage
Source: Transl Neurosci. 2026 Jun 8;17(1):20250394. doi: 10.1515/tnsci-2025-0394 (PMC13241177; doi:10.1515/tnsci-2025-0394)
Supplement: Supplementary file 2 — Supplementary Material [file j_tnsci-2025-0394_suppl_002.docx]

Supplementary Figure 1. Bioinformatics analysis. (A) ENCORI (https://rnasysu.com/encori/) analyzed the binding of FTO to PDCD5; (B) SRAMP (http://www.cuilab.cn/sramp) showed the m6A binding sites on PDCD5.
